# *GSF2* deletion increases lactic acid production by alleviating glucose repression in *Saccharomyces cerevisiae*

**DOI:** 10.1038/srep34812

**Published:** 2016-10-06

**Authors:** Seung-Ho Baek, Eunice Y. Kwon, Seon-Young Kim, Ji-Sook Hahn

**Affiliations:** 1 School of Chemical and Biological Engineering, Institute of Chemical Processes, Seoul National University, 1 Gwanak-ro, Gwanak-gu, Seoul 08826, Republic of Korea; 2 Personalized Genomic Medicine Research Center, KRIBB, 125 Gwahag-ro, Yuseong-gu, Daejeon 34141, Republic of Korea

## Abstract

Improving lactic acid (LA) tolerance is important for cost-effective microbial production of LA under acidic fermentation conditions. Previously, we generated LA-tolerant D-LA-producing *S. cerevisiae* strain JHY5310 by laboratory adaptive evolution of JHY5210. In this study, we performed whole genome sequencing of JHY5310, identifying four loss-of-function mutations in *GSF2*, *SYN8*, *STM1*, and *SIF2* genes, which are responsible for the LA tolerance of JHY5310. Among the mutations, a nonsense mutation in *GSF2* was identified as the major contributor to the improved LA tolerance and LA production in JHY5310. Deletion of *GSF2* in the parental strain JHY5210 significantly improved glucose uptake and D-LA production levels, while derepressing glucose-repressed genes including genes involved in the respiratory pathway. Therefore, more efficient generation of ATP and NAD^+^ via respiration might rescue the growth defects of the LA-producing strain, where ATP depletion through extensive export of lactate and proton is one of major reasons for the impaired growth. Accordingly, alleviation of glucose repression by deleting *MIG1* or *HXK2* in JHY5210 also improved D-LA production. *GSF2* deletion could be applied to various bioprocesses where increasing biomass yield or respiratory flux is desirable.

Microbial production of lactic acid (LA) has received a great attention for the production of poly lactic acid (PLA), a biodegradable polymer^1^,^2^,^3^. Lactic acid bacteria naturally produce LA, but neutralizing reagent such as CaCO_3_ should be added during fermentation due to their acid sensitivity. Such a neutralizing fermentation process requires recovery of LA from the resulting calcium salt of lactate by treating sulfuric acid, producing gypsum as an undesirable byproduct^4^,^5^,^6^. Therefore, *Saccharomyces cerevisiae* having higher acid tolerance than lactic acid bacteria is considered a promising host for LA production^5^,^6^,^7^,^8^,^9^. However, even in *S. cerevisiae*, growth inhibition caused by LA accumulation is the major limiting factor preventing high-titer production of LA^7^,^8^. Under acidic conditions, undissociated LA molecules in the medium diffuse through the plasma membrane and dissociate into the acid anions and protons in the cytosol, where the pH is neutral. This leads to growth-inhibitory stress conditions including cytosolic acidification, modifications of cellular components, and energy depletion from the excessive use of ATP to export protons and lactate ions^5^,^6^,^8^,^10^,^11^. Therefore, to produce LA without neutralization during fermentation, it is critical to improve LA tolerance.

Cellular responses to weak acids such as acetic acid, lactic acid, benzoic acid, and sorbic acid are variable depending on the chemical properties of weak acids^12^,^13^. Transcriptome analyses in *S. cerevisiae* have revealed that Aft1 transcription factor plays an important role in induction of genes involved in iron homeostasis in the presence of lactate anion, which might reflect iron chelating activity of lactate^10^,^14^. On the other hand, Haa1 transcriptional activator is mainly responsible for cellular response to undissociated lactic acid^10^,^15^. Accordingly, we previously showed that overexpression of Haa1 could improve LA tolerance and LA production under acidic fermentation conditions^16^. Genome-wide screening of nonessential deletion strain collection or RNAi-mediated knockdown library discovered several genes whose deletion or knockdown could improve LA tolerance^9^,^14^,^17^. The identified genes cover a wide range of biological functions such as cell wall components, histone acetyltransferase complex, and a ribosome-associated chaperone, implying the complexity of cellular defense mechanisms against LA stress. LA tolerance can be improved by logical genetic modifications based on the stress-tolerance mechanisms^6^,^18^. However, considering the fact that LA tolerance mechanisms are not fully understood and involve complex networks of multiple genes^15^, adaptive laboratory evolution is another efficient strategy to obtain tolerant strains^19^,^20^. This can be a powerful tool in combination with whole genome sequencing analysis and reverse metabolic engineering for the identification of modified genes and pathways, which are difficult to predict rationally.

In this study, we identified genes involved in LA tolerance from genome sequencing of LA-tolerant strain JHY5310, generated by adaptive evolution in our previous study. We demonstrated that alleviating glucose repression by *GSF2* deletion can significantly improve LA tolerance and LA production possibly by eliciting more efficient ATP synthesis via respiratory pathway.

## Results

### Whole genome sequencing analysis of LA-tolerant strain JHY5310

**Figure 1:**
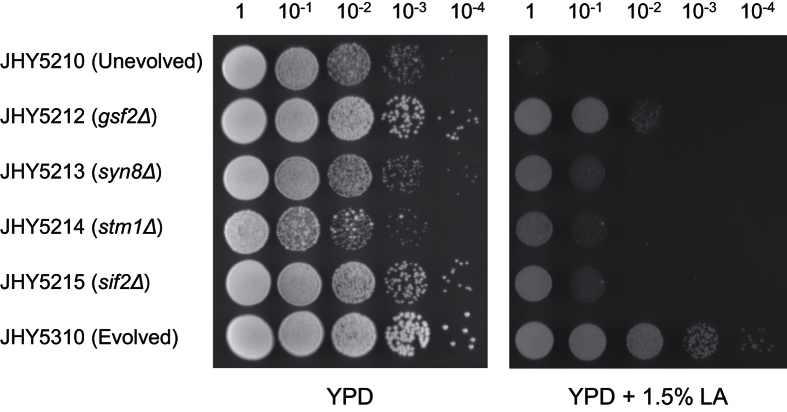
Identification of genes responsible for LA tolerance of JHY5310. Unevolved parental strain JHY5210, deletion strains derived from JHY5210, and evolved strain JHY5310 were grown in YPD medium and then OD_600_ of 1 cells were serially diluted and spotted onto YPD solid medium with or without 1.5% LA.

Previously, we generated D-LA-producing *S. cerevisiae* strain JHY5210 (*dld1Δjen1Δadh1Δgpd1Δgpd2Δpdc1Δ::Lm.ldhA*) by expressing D-lactate dehydrogenase gene (*ldhA*, LEUM_1756) from *Leuconostoc mesenteroides* subsp. *mesenteroides* ATCC 8293 and by deleting *DLD1* encoding D-lactate dehydrogenase, *JEN1* encoding monocarboxylate transporter, and major competing pathways producing ethanol and glycerol^16^. In addition, from adaptive laboratory evolution of the strain JHY5210, we isolated strain JHY5310 with improved LA tolerance (Fig. 1). JHY5310 also showed improved growth even in the absence of LA in the medium (Fig. 1).

To identify genes involved in LA tolerance in JHY5310, whole genome sequencing of JHY5310 and its parental strain JHY5210 was carried out. In comparison with JHY5210, mutations in *GSF2*, *SYN8*, *STM1*, and *SIF2* genes were identified in JHY5310 (Table 1). *GSF2* in the evolved strain has a novel stop codon by point mutation at position 4, immediately after start codon. A nonsense mutation was also found in *SYN8*, which changes the codon for Glu121 to stop codon. *STM1* gene has a 17-bp internal deletion from position 417 to 433, resulting in a frameshift mutation after Asp140. A missense mutation was found in *SIF2*, resulting in Met to Ile substitution at 66 amino acid residue.

Gsf2, an integral membrane protein localized in the endoplasmic reticulum (ER), is known to be involved in maturation and secretion of certain type of hexose transporters such as Hxt1 and Gal2 to the plasma membrane^21^,^22^,^23^. Syn8 is an endosomal SNARE protein, and deletion of *SYN8* was previously reported to increase LA tolerance^9^,^14^. Stm1, a protein required for facilitating translation under nutrient stress condition, is known to be associated with apoptosis and telomere biosynthesis^24^,^25^. Sif2 encodes a component of Set3C histone deacetylase complex^26^. Any gene duplication or insertion was not found in the evolved strain JHY5310.

### Effects of mutated genes on LA tolerance and LA production

The identified nonsense mutations (*GSF2* and *SYN8*) and a frameshift mutation (*STM1*) might be loss-of-function mutations. Therefore, we first investigated the effects of deleting the mutated genes on LA tolerance. *GSF2*, *SYN8*, *STM1*, and *SIF2* genes were deleted in the parental strain JHY5210, and their growth was compared on YPD solid medium in the presence or absence of 1.5% LA (Fig. 1). All deletion strains showed enhanced LA tolerance compared to JHY5210, although to a less extent than the evolved strain JHY5310. Among the four genes, deletion of *GSF2* was most effective in enhancing LA tolerance. These results suggest that the identified four mutations might be loss-of-function mutations that all contribute to the LA tolerance of JHY5310. On the other hand, the deletion strains showed different growth rates on control YPD medium without LA. Compared to JHY5210, JHY5212 (*gsf2Δ*) and JHY5215 (*sif2Δ*) showed improved cell growth, whereas JHY5214 (*stm1Δ*) showed a slight growth defect under normal conditions (Fig. 1). Therefore, deletion of *GSF2* and *SIF2* might improve cellular fitness of JHY5210, whereas the deletion effects of *SYN8* and *STM1* might be more specific to cellular defense against LA stress.

**Figure 2:**
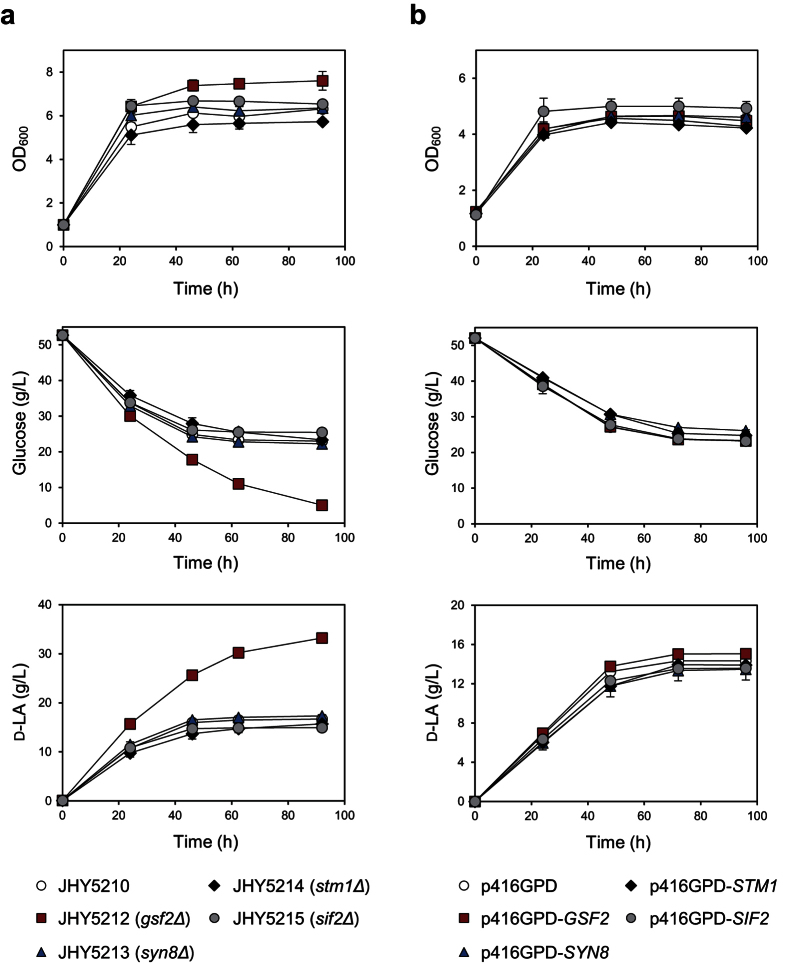
Effects of gene deletion or overexpression on D-LA production and glucose consumption levels. (**a**) Indicated strains were cultured in YPD medium containing 50 g/L glucose. Cell growth and glucose and D-LA levels in the medium were detected. **(b)** JHY5210 cells harboring the indicated plasmid were grown in SC-Ura medium containing 50 g/L glucose. Cells harboring p416GPD plasmid were used as a control. Error bars indicate standard deviations of three independent experiments.

Next, we examined the effect of each gene on LA production. Although all deletion strains showed enhanced LA tolerance, only JHY5212 showed significantly higher glucose consumption and LA production levels compared to JHY5210 (Fig. 2a), which is consistent with the biggest effect of *GSF2* deletion on LA tolerance (Fig. 1). We also tested the effects of overexpressing the identified genes. None of these genes showed any significant improvement of glucose consumption and D-LA production when overexpressed, supporting the idea that LA tolerance is the result of inactivation of these genes in JHY5310 (Fig. 2b).

### *GSF2* deletion improved LA production by alleviating glucose repression

**Figure 3:**
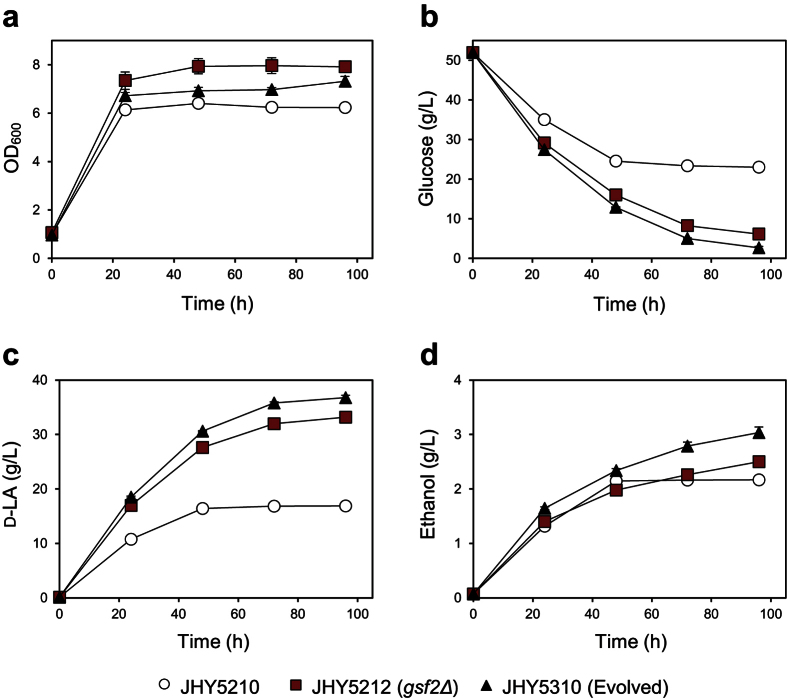
Effects of *GSF2* deletion on D-LA production and glucose consumption levels. Cells were grown in YPD medium containing 50 g/L glucose. Cell growth **(a)**, residual glucose concentrations **(b)**, D-LA **(c)**, and ethanol **(d)** production levels in the medium were monitored. Error bars indicate standard deviations of three independent experiments.

Since strain JHY5212 showed the best performance in LA production, we compared this strain with the evolved strain JHY5310. When the unevolved strain JHY5210 was cultured in YPD medium containing 50 g/L glucose, only 28.9 g/L glucose was consumed, producing 16.9 g/L D-LA with a yield of 0.58 g/g glucose (Fig. 3). Medium pH dropped from 6.6 to 3.2 during the fermentation (Supplementary Figure S1), supporting the idea that acidification of the medium might be a critical growth inhibitory factor. On the other hand, the evolved strain JHY5310 consumed 49.3 g/L of glucose, producing 36.8 g/L D-LA with a yield of 0.75 g/g glucose (Fig. 3). JHY5212 showed slightly lower glucose consumption (45.8 g/L) and D-LA production (33.2 g/L) levels than did JHY5310 (Fig. 3). Taken together, these results suggest that the nonsense mutation of *GSF2* is the major contributor to the enhanced LA tolerance and LA production in the evolved strain JHY5310, with minor contributions of 3 other mutations identified in the genome.

**Figure 4:**
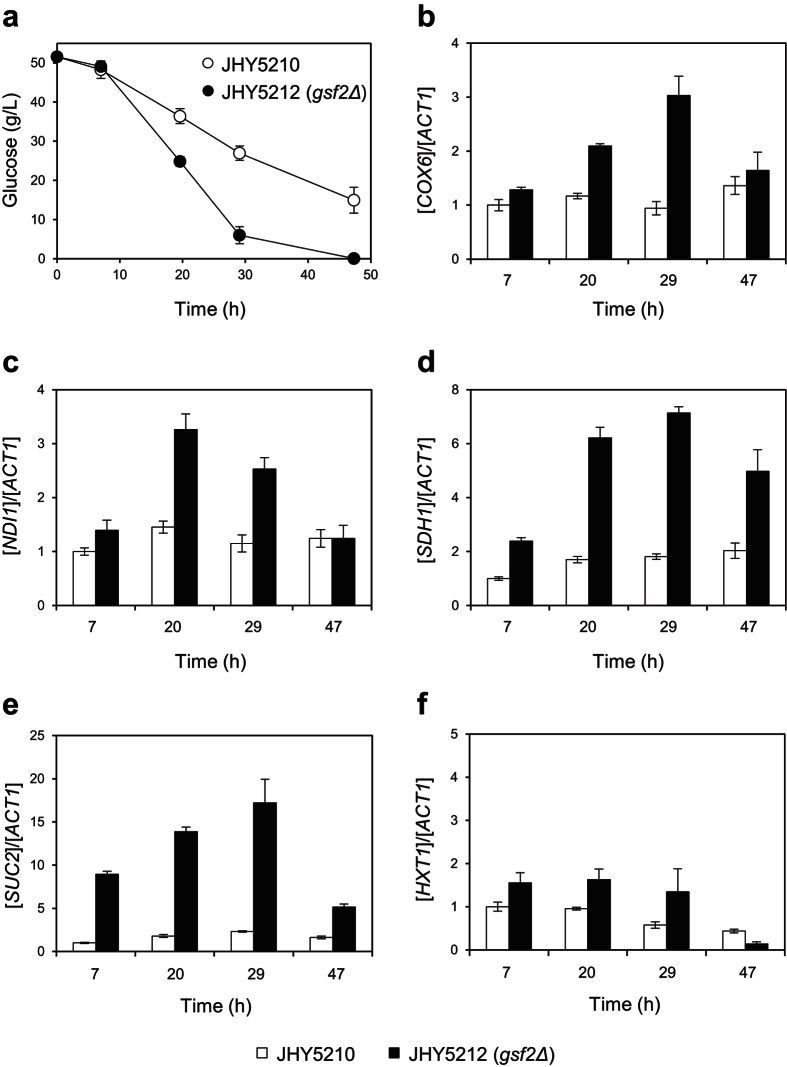
Derepression of glucose-repressed genes by *GSF2* deletion. JHY5210 and JHY5212 strains were grown in YPD medium containing 50 g/L glucose and residual glucose concentrations were measured (**a**) and mRNA levels of *COX6* (**b**), *NDI1* (**c**), *SDH*1 (**d**), *SU*C2 (**e**), and *HXT1* (**f**) genes were quantified by qRT-PCR normalized with *ACT1* mRNA levels. Error bars indicate standard deviations of three independent experiments.

Gsf2, an ER membrane protein, is known to be involved in the transportation of a subset of hexose transporters such as Hxt1 to the plasma membrane^21^,^22^,^23^. Therefore, *GSF2* deletion leads to a decrease in functional localization of Hxt1 in the plasma membrane, resulting in reduced glucose uptake rate, which in turn can alleviate glucose repression^21^. In *S. cerevisiae*, glucose repression is the major regulatory mechanism of deriving high glucose flux toward ethanol fermentation while inhibiting respiratory growth even under aerobic conditions^27^,^28^,^29^. Therefore, considering the fact that ATP depletion caused by extensive use of H^+^-ATPase and efflux pumps to export protons and lactate anions is one of the major reasons for growth inhibition upon accumulation of LA, more efficient ATP synthesis through respiration might be responsible for the improved growth of JHY5212 during LA production. In addition, since ethanol and glycerol production pathways were largely blocked in JHY5210, NAD^+^ regeneration via heterologous lactate dehydrogenase might not be sufficient for cell growth, and an increase in respiration might rescue the defect of NAD^+^ regeneration. In agreement with this idea, JHY5212 showed higher mRNA levels of *COX6, NDI1*, and *SDH1* genes involved in the mitochondrial respiratory chain and TCA cycle than did JHY5210 (Fig. 4b–d). *SUC2*, another glucose-repressed gene, was also highly expressed in JHY5212 even in the presence of high level of glucose in the medium (Fig. 4e), supporting the proposed effect of *GSF2* deletion on glucose derepression. In addition, expression of *HXT1*, which is also regulated by glucose repression^30^, was slightly increased by *GSF2* deletion (Fig. 4f), suggesting that the effect of *GSF2* deletion on glucose derepression is not due to a reduced transcription level of *HXT1*. In contrast, in JHY5210, glucose-repressed genes were not derepressed until 47 h, suggesting that ATP synthesis via respiration might not be sufficient to circumvent ATP depletion.

### Glucose derepression by deleting *MIG1* or *HXK2* improved LA production

**Figure 5:**
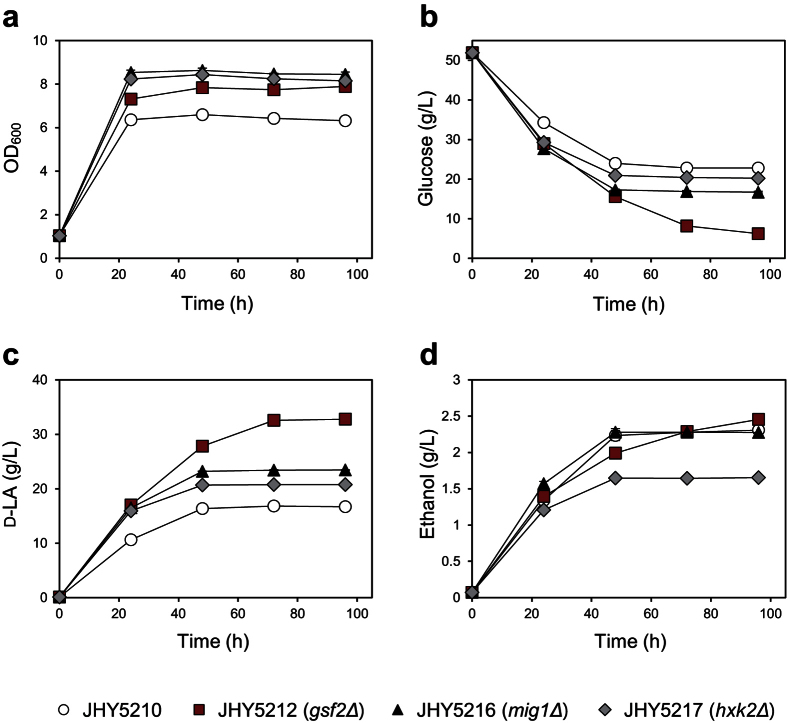
Improvement of D-LA production by deleting *HXK2* or *MIG1* involved in glucose repression. JHY5210 and JHY5210-derived deletion strains were cultured in YPD medium containing 50 g/L glucose. Cell growth (**a**), residual glucose concentration (**b**), production levels of D-LA (**c**) and ethanol (**d**) were monitored. Error bars indicate standard deviations of three independent experiments.

Based on the hypothesis that *GSF2* deletion in JHY5210 might increase LA production by relieving glucose repression, we tested whether other genetic modifications known to relieve glucose repression could also increase LA production. Mig1 is a well-known transcriptional repressor of the glucose-repressed genes. Hxk2 is a major cytosolic hexokinase involved in phosphorylation of glucose, but is also involved in transcriptional repression of glucose-repressed genes in the nucleus by interacting with Mig1^27^,^28^,^29^. Both Mig1 and Hxk2 are negatively regulated by Snf1 kinase, an AMP-activated Ser/Thr kinase playing a central role in glucose derepression. Deletion of either *MIG1* or *HXK2* is known to alleviate glucose repression^31^. Therefore, we deleted *MIG1* or *HXK2* in JHY5210, and tested for LA production capability. In agreement with our hypothesis, deletion of *MIG1* or *HXK2* led to increased glucose uptake and LA production levels compared to JH5210 (Fig. 5). However, the positive effect of *MIG1* or *HXK2* deletion on LA production was weaker than that of *GSF2* deletion. Taken together, increasing glucose flux to respiratory pathway by alleviating glucose repression might be beneficial to improve LA production in JHY5210.

### *GSF2* deletion in wild-type strain showed metabolic phenotypes of increased respiration

**Figure 6:**
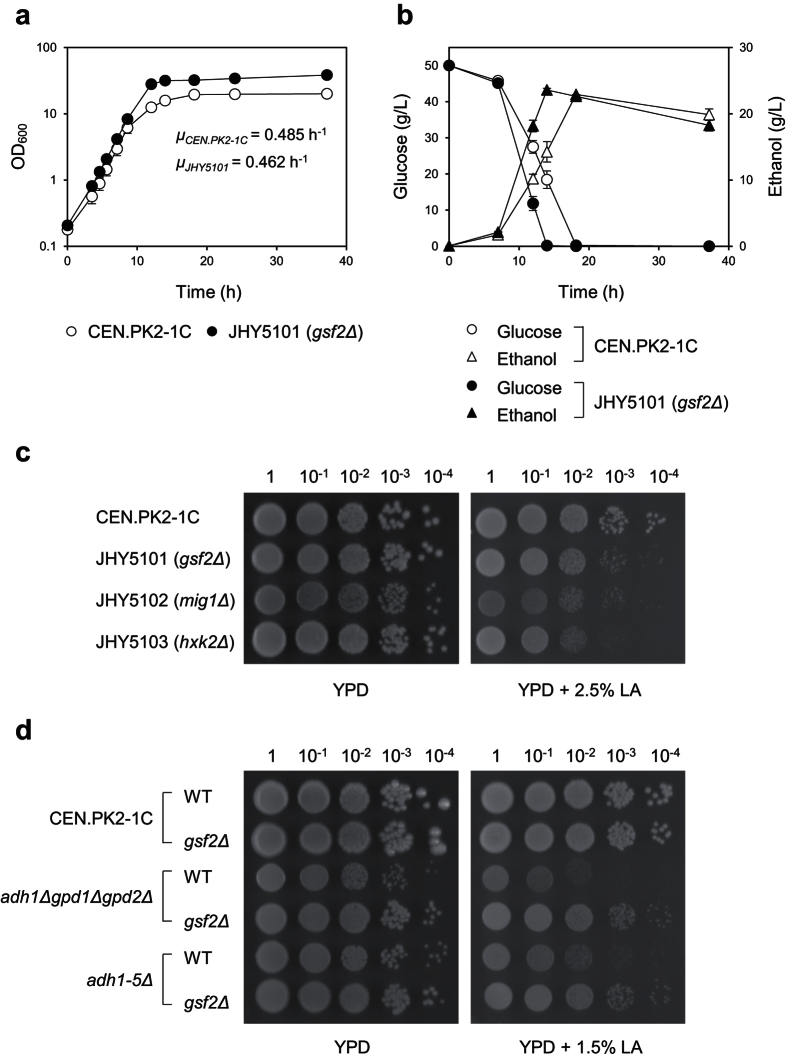
Increasing respiration capability by *GSF2* deletion in wild type (CEN.PK2-1C). Cell growth (**a**) and metabolite profiles (**b**) of CEN.PK2-1C and JHY5101 were compared during growth in YPD medium containing 50 g/L glucose. (**c**) To test LA tolerance, the indicated deletion strains derived from CEN.PK2-1C were grown in YPD medium and then OD_600_ of 1 cells were serially diluted and spotted onto YPD solid medium with or without 2.5% LA. (**d**) The effect of *GSF2* deletion in different strain backgrounds were monitored on YPD medium with or without 1.5% LA.

Next, we investigated the effects of *GSF2* deletion on cell growth of wild-type strain. *GSF2* deletion in wild-type strain CEN.PK2-1C (strain JHY5101) led to a slight, but significant (*P *< 0.05) decrease in specific growth rate (*μ*_JHY5101_ = 0.462 ± 0.004 h^−1^) compared to wild type (*μ*_CEN.PK2-1C_ = 0.485 ± 0.010 h^−1^), but increased final cell density (Fig. 6a), consistent with the fact that increasing respiratory capacity increases the biomass yield^32^. In *S. cerevisiae*, specific glucose consumption rate shows a positive correlation with specific ethanol production rate (fermentation capability)^33^. During the exponential growth phase, *GSF2* deletion led to a 26% decrease in specific glucose consumption rate (*q*_*glucose*_ = 5.31 ± 0.07 mmol∙h^−1^∙g^−1^ of dry biomass) and a 24% decrease in specific ethanol production rate (*q*_*ethanol*_ = 10.01 ± 0.11 mmol∙h^−1^∙g^−1^ of dry biomass) compared to the parental strain CEN.PK2-1C (*q*_*glucose*_ = 7.22 ± 0.51 mmol∙h^−1^∙g^−1^ of dry biomass and *q*_*ethanol*_ = 13.11 ± 0.72 mmol∙h^−1^∙g^−1^ of dry biomass) (Fig. 6b). These results further support the hypothesis that the reduced specific glucose uptake rate in *GSF2* deletion strain increases glucose flux to respiration by relieving glucose repression.

### *GSF2* deletion restores growth defects caused by insufficient NAD^+^ regeneration

We also tested whether *GSF2* deletion in wild-type strain can increase LA tolerance. In contrast to the result observed in the LA-producing strain JHY5210, *GSF2* deletion in wild type CEN.PK2-1C reduced LA tolerance on medium containing 2.5% LA (Fig. 6c). LA sensitivity was also observed in *MIG1* and *HXK2* deletion strains, implying that LA sensitivity might be a common phenotype of the glucose derepressed cells with increased respiration capacity (Fig. 6c). These results suggest that *GSF2* deletion might increase LA tolerance in JHY5210 by rescuing its growth defects. Note that JHY5210 is much more sensitive to LA than CEN.PK2-1C. JHY5210 barely survived on YPD medium containing 1.5% LA (Fig. 1), whereas CEN.PK2-1C grew normally on the same medium (Fig. 6d).

In addition to the endogenously produced LA, insufficient NAD^+^ regeneration caused by blocking ethanol production pathway (*adh1Δpdc1Δ*) and glycerol production pathway (*gpd1Δgpd2Δ*) might contribute to the growth defects of JHY5210. Therefore, we investigated whether the growth defects of strains with impaired NAD^+^ regeneration, but without LA production, can be rescued by *GSF2* deletion. JHY602 (*adh1-5Δ*) lacking five genes encoding alcohol dehydrogenase (ADH) and JHY604 (*adh1Δgpd1Δgpd2Δ*) having deletions of major ADH gene (*ADH1*) and genes involved in glycerol production (*GPD1* and *GPD2*) have growth defects due to the insufficient NAD^+^ regeneration. The growth defects of these strains on YPD medium were restored by *GSF2* deletion (Fig. 6d). In particular, the growth rescuing effect of *GSF2* deletion became more prominent in the presence LA in the medium (Fig. 6d), which might imply that respiration-dependent increase in ATP synthesis might be more critical for the survival of JHY602 and JHY604 under the conditions requiring higher ATP to circumvent LA toxicity.

Taken together, the effects of *GSF2* deletion on LA tolerance might be dependent on the cellular metabolic status. For the cells having growth defects due to the impaired NAD^+^ regeneration or insufficient ATP synthesis, *GSF2* deletion might contribute to improve LA tolerance.

## Discussion

Increasing LA tolerance is one of the important issues to improve LA production under acidic fermentation conditions^6^,^8^,^9^. In our previous study, we enhanced LA tolerance of a D-LA-producing *S. cerevisiae* strain JHY5210 by using adaptive laboratory evolution^16^. In this study, we carried out whole genome sequencing analysis of the evolved strain JHY5310, followed by functional studies to identify mutated genes responsible for LA tolerance. We identified loss-of-function mutations in *GSF2*, *SYN8*, *STM1*, and *SIF2* genes, which contribute to the LA tolerance of JHY5310. Among the four genes, deletion of *GSF2* in the parental strain JHY5210 largely mimicked the LA tolerance and LA production properties of strain JHY5310. Gsf2 is known to be necessary for proper localization of certain hexose transporters including Hxt1 in the plasma membrane^21^,^22^,^23^. Therefore, deletion of *GSF2* leads to reduced glucose uptake rate, thereby alleviating glucose repression. *S. cerevisiae* is a Crabtree positive strain having a strong tendency of catabolizing glucose via ethanol fermentation even under aerobic conditions^34^,^35^. Therefore, targets of glucose repression include not only gluconeogenesis and utilization of alternative carbon sources, but also respiration^27^,^29^. We confirmed that *GSF2* deletion derepressed glucose-repressed genes including genes involved in respiratory pathway. More efficient ATP synthesis and NAD^+^ regeneration by respiration might rescue the growth defects of JHY5210. Considering the fact that ATP depletion via intensive export of protons and lactate anions is one of the major factors inhibiting cell growth in LA-producing cells^5^,^6^,^8^,^10^,^11^, more efficient ATP synthesis via respiration might contribute to the LA tolerance in JHY5212 lacking *GSF2*.

However, *GSF2* deletion in wild-type strain led to an opposite effect of increasing LA sensitivity, which was commonly observed in other glucose derepressed mutants such as *mig1Δ* and *hxk2Δ*. Therefore, unlike in JHY5210 strain, LA-dependent depletion of ATP might play a minor role in LA toxicity in wild type. LA-dependent depletion of ATP might be more detrimental for cells with impaired energy metabolism. Instead, increasing respiration by glucose derepression seems to increase LA sensitivity in wild-type background, which might be related to the fact that LA can induce oxidative stress, which is linked to the generation of reactive oxygen species (ROS) during the respiratory metabolism^18^,^36^. These results are consistent with a previous report showing higher LA toxicity under aerobic conditions than anaerobic conditions^18^.

In *S. cerevisiae*, glucose uptake is facilitated by hexose transporters (HXTs), Hxt1 to Hxt7, in the plasma membrane^30^. HXTs, having different glucose affinities, are differentially expressed depending on extracellular glucose concentrations by complex regulatory networks involving both glucose induction and glucose repression mechanisms^30^. Glucose induction is mainly mediated by plasma membrane glucose sensors, Snf3 and Rgt2, and transcriptional repressor Rgt1^37^, whereas Snf1 kinase and Mig1 repressor plays a central role in glucose repression^27^,^29^,^38^. In the absence of glucose, expression of *HXT1*, *HXT2*, *HXT3*, and *HXT4* genes encoding low to medium-affinity (*K*_*m*_~5–100 mM) glucose transporters are repressed by Rgt1 in association with co-repressors Mth1 and Std1^27^,^39^. The presence of extracellular glucose was sensed by Snf3 and Rgt2, leading to the degradation of Mth1 and Std1, which then resulted in derepression of *HXT* genes^38^. In addition, Mig1 represses *MTH1* expression under high-glucose conditions, reinforcing the inactivation of Mth1 by glucose^27^. On the other hand, expression of *HXT1*, *HXT2*, *HXT3*, and *HXT4* as well as *HXT6* and *HXT7* encoding high-affinity (*K*_*m*_~1 mM) glucose transporters is repressed by Mig1 in the presence of glucose^30^. Mig1 also represses other glucose-repressed genes in association with Hxk2. Hxk2 has a dual role as a major hexokinase under high-glucose conditions and as an intracellular glucose sensor positively regulating glucose repression^40^. Upon glucose depletion, Snf1 is activated, which in turn inactivates Mig1 and activates transcription factors such as Cat8, Sip4, and Adr1, resulting in expression of genes involved in the utilization of alternative carbon sources, gluconeogenesis, glyoxylate cycle, and respiration^29^,^38^. We showed that alleviating glucose repression by *GSF2* deletion is more effective in increasing LA tolerance than the deletion of *MIG1* or *HXK2*. These results might be in part related to the fact that Mig1 and Hxk2 regulate only a subset of glucose repressed genes, whereas the decrease in glucose uptake rate in *gsf2Δ* might trigger wider range of cellular responses.

Although rapid glucose uptake by efficient ethanol fermentation might provide a selective advantage for yeast cells living in natural environments, glucose repression of the respiratory pathway might be undesirable for some industrial applications. For example, in the case of producing biomass-directed products such as cell itself or proteins, it might be useful to shift the metabolic flux from fermentation to respiration, which can provide higher biomass yield on glucose^32^,^41^. On the other hand, to maximize metabolite production, it is desirable to minimize biomass yield. However, depending on the properties of target chemicals and engineered biosynthetic pathways, increasing respiration capacity by alleviating glucose repression can be advantageous to improve production. In this study, we demonstrated one such example that more efficient generation of ATP and NAD^+^ via respiration could rescue the growth defects of LA-producing cells. In addition, more efficient regenerating NAD^+^ through respiratory pathway could be desirable for engineered strains having redox imbalance. For example, relieving glucose repression restored the growth defects of *S. cerevisiae* strain lacking pyruvate decarboxylase (PDC)^42^,^43^,^44^. PDC catalyzes the conversion of pyruvate to acetaldehyde, the first step in the ethanol production pathway. Therefore, PDC-negative strain (*pdc1Δ*, *pdc5Δ*, and *pdc6Δ*) could be useful as a platform strain to produce pyruvate-derived products, but its practical applications are limited due to severe growth defects in high glucose and requirement of C_2_ such as ethanol and acetate^45^. Evolved PDC-negative strains developed by three different groups revealed mutations in the same gene, *MTH1*^42^,^43^,^44^. The identified Mth1 mutants are supposed to be resistant to glucose-dependent degradation, resulting in reduced glucose influx by repressing *HXT* genes. In the case of PDC-negative strain, glucose derepression might rescue the growth defects by preventing intracellular accumulation of pyruvate to toxic levels and by regeneration of NAD^+^ via respiration^42^. Introduction of the *MTH1* mutant genes into the PDC-negative strain has been applied to produce pyruvate, LA, and 2,3-butandiol^44^,^46^,^47^.

Impaired plasma membrane localization of Hxt1, the major HXT expressed under high-glucose conditions, might be the main reason for the glucose derepression phenotype of *GSF2* deletion strain, but proper localization of other membrane proteins might also require Gsf2 function^21^. Therefore, we cannot rule out the possibility that impaired localization of other Gsf2 target proteins might also contribute to the LA tolerance of *GSF2* deletion strain. However, since deletion of *GSF2* has been proven to be effective in inducing glucose derepression in different strain backgrounds including wild type, it could be applied to various applications where increasing biomass yield or respiratory flux is desirable. In addition to producing biomass-directed products, *GSF2* deletion would be useful if the production of target chemicals requires efficient ATP production or NAD^+^ regeneration, and also for the production of TCA cycle intermediates such as succinic acid and fumaric acid.

## Methods

### Strains and media and culture conditions

All yeast strains and primers used in this study are listed in Table 2 and Supplementary Table S1, respectively. *S. cerevisiae* CEN.PK2-1C strain (*MAT***a** *ura3-52 trp1-289 leu2-3,112 his3Δ1 MAL2-8C SUC2*) was used as a parental strain. Deletion strains were generated by PCR-mediated homologous recombination based on Cre/loxP recombination system^48^. Deletion cassettes were obtained by PCR amplification from pUG27 or pUG72 plasmid using target gene-specific primer pairs (d_GENE F and d_GENE R) and then introduced into *S. cerevisiae* strains. Correct integration of the cassette was confirmed by PCR analysis using confirmation primer pairs (c_GENE F and c_GENE R). All yeast cells were cultured in YPD medium (20 g/L peptone, 10 g/L yeast extract, and 20 or 50 g/L glucose) or synthetic complete (SC) medium (6.7 g/L yeast nitrogen base without amino acids, 20 or 50 g/L glucose, 1.67 g/L amino acids dropout mixture lacking His, Trp, Leu, and Ura) supplemented with auxotrophic amino acids as required. For LA production experiments, OD_600_ of 1 of pre-cultured yeast strains were harvested and resuspended in 5 mL of YPD or SC-Ura medium containing 50 g/L glucose. Yeast cells were cultured in a 50 mL screw cap conical tube at 30 °C with shaking at 170 rpm.

### Plasmids

Plasmids used in this study are listed in Table 2. *GSF2*, *SYN8*, *STM1*, and *SIF2* ORFs were amplified by PCR from CEN.PK2-1C genomic DNA, and then cloned between BamHI and SalI sites of p416GPD plasmid.

### Whole genome sequencing analysis

The unevolved strain JHY5210 and the evolved strain JHY5310 were cultured and harvested for the genomic DNA extraction. All genomic DNA were isolated using HiGeneTM Genomic DNA Prep Kit for Yeast (BIOFACT, Korea). The DNA library was prepared using the TruSeq DNA sample preparation kits (Illumina, USA) and sequenced using Illumina Hiseq-2000 (Illumina, USA) at 2 × 75 bp read pairs. The raw reads were processed with Trimmomatic (version 0.3) to remove adapters and poor quality reads, and then reads shorter than 36 bp were discarded. The filtered reads were mapped to the reference genome (CEN.PK 113-7D, http://cenpk.tudelft.nl) using Burrows-Wheeler Aligner (BWA) software (ver 0.7.1). Potential PCR duplicates were removed with the MarkDuplicates program of the Picard package (http://picard.sourceforge.net). Indels were located and realigned with Realigner Target Creator/Indel Realigner of Genome Analysis Toolkit (GATK), and detected using VarScan software (ver. 2.3.7). Single nucleotide variants (SNVs) were detected using MuTect (ver 1.1.7).

### Quantitative reverse transcription PCR (qRT-PCR) analysis

OD_600_ of 0.5 of JHY5210 and JHY5212 strains were cultured in 10 ml YPD medium containing 50 g/L glucose in a 100 ml flask at 30 °C for 47 h. Total RNA from each harvested cells was isolated using the hot acidic phenol RNA extraction method^49^, and then the relative amount of mRNA was determined by qRT-PCR^50^ using a LightCycler 480 II instrument (Roche Diagnostics, Germany) with SYBR green PCR master mix (Roche Diagnostics, Germany). The *ACT1* gene was used as a control to normalize the transcription level of target genes. Primer sequences used for qRT-PCR are listed in Supplementary Table S1.

### Analytical methods

Samples collected from culture supernatant were filtered using a 0.22 μm syringe filter before detecting metabolites. To quantify the concentration of ethanol, glucose and lactate, high performance liquid chromatography (HPLC) analysis was performed in UltiMate 3000 HPLC system (Thermo Fishers Scientific) equipped with Bio-Rad Aminex HPX-87H column (300 mm × 7.8 mm, 5 μm) at 60 °C with 5 mM H_2_SO_4_ at a flow rate of 0.6 mL/min. Refractive index (RI) was used as a detector keeping at 35 °C. Growth was monitored by determining optical density at 600 nm using spectrophotometer (Varian Cary50 UV/Vis spectrophotometer, Agilent) as described previously. Specific rates of glucose consumption and ethanol formation (*q*: mmol∙h^−1^∙g^−1^ of dry biomass) in the exponential growth phase were calculated from three independent experiments. Dry cell weight (DCW) was estimated by multiplying optical density value and a conversion factor (0.3) together^51^.

## Additional Information

**How to cite this article**: Baek, S.-H. *et al*. *GSF2* deletion increases lactic acid production by alleviating glucose repression in *Saccharomyces cerevisiae*. *Sci. Rep*. **6**, 34812; doi: 10.1038/srep34812 (2016).

## Supplementary Material

Supplementary Information

## Figures and Tables

**Table 1:** Mutations in the JHY5310 genome.

Gene	Type of mutation	Nucleotide change	Amino acid change
*GSF2*	Nonsense (GAG → TAG)	4G → T	Glu2 → Stop
*SYN8*	Nonsense (GAG → TAG)	361G → T	Glu121 → Stop
*STM1*	Frameshift	Δ417–433	Frameshift after Asp140
*SIF2*	Missense (ATG → ATT)	198G → T	Met66 → Ile

**Table 2:** Yeast strains and plasmids used in this study.

Strain / Plasmid	Genotype and description	Reference
*S. cerevisiae* strains		
CEN.PK2-1C	*MAT**a** ura3-52 trp1-289 leu2-3,112 his3Δ1 MAL2-8C SUC2*	EUROSCARF
JHY5101	CEN.PK2-1C *gsf2Δ::loxP-URA3-loxP*	This study
JHY5102	CEN.PK2-1C *mig1Δ::loxP-URA3-loxP*	This study
JHY5103	CEN.PK2-1C *hxk2Δ::loxP-his5*^+^*-loxP*	This study
JHY5160	CEN.PK2-1C *dld1Δ::loxP jen1Δ::loxP adh1Δ::loxP gpd1Δ::loxP gpd2Δ::loxP*	16
JHY5210	JHY5160 *pdc1Δ::*P_*TEF1*_-*Lm.ldhA*-T_*CYC1*_	16
JHY5212	JHY5210 *gsf2Δ::loxP-his5*^+^*-loxP*	This study
JHY5213	JHY5210 *syn8Δ::loxP-his5*^+^*-loxP*	This study
JHY5214	JHY5210 *stm1Δ::loxP-his5*^+^*-loxP*	This study
JHY5215	JHY5210 *sif2Δ::loxP-his5*^+^*-loxP*	This study
JHY5216	JHY5210 *mig1Δ::loxP-his5*^+^*-loxP*	This study
JHY5217	JHY5210 *hxk2Δ::loxP-his5*^+^*-loxP*	This study
JHY5310	Evolved strain from JHY5210	16
JHY602	CEN.PK2-1C *adh1Δ::loxP adh2Δ::loxP adh3Δ::loxP adh4Δ::loxP adh5Δ::loxP*	52
JHY5401	JHY602 *gsf2Δ::loxP-URA3-loxP*	This study
JHY604	CEN.PK2-1C *adh1Δ::loxP gpd1Δ::loxP gpd2Δ::loxP*	52
JHY5402	JHY604 *gsf2Δ::loxP-URA3-loxP*	This study
Plasmids		
pUG27	Plasmid containing *loxP-his5*^+^*-loxP* deletion cassette	EUROSCARF
pUG72	Plasmid containing *loxP-URA3-loxP* deletion cassette	EUROSCARF
p416GPD	CEN/ARS plasmid, *URA3*, P_*TDH3*_, T_*CYC1*_	53
p416GPD-*GSF2*	CEN/ARS plasmid, *URA3*, P_*TDH3*_-*GSF2*-T_*CYC1*_	This study
p416GPD-*SYN8*	CEN/ARS plasmid, *URA3*, P_*TDH3*_-*SYN8*-T_*CYC1*_	This study
p416GPD-*STM1*	CEN/ARS plasmid, *URA3*, P_*TDH3*_-*STM1*-T_*CYC1*_	This study
p416GPD-*SIF2*	CEN/ARS plasmid, *URA3*, P_*TDH3*_-*SIF2*-T_*CYC1*_	This study
