# Supplementary material for: GSF2 deletion increases lactic acid production by alleviating glucose repression in Saccharomyces cerevisiae
Source: Sci Rep. 2016 Oct 6;6:34812. doi: 10.1038/srep34812 (PMC5052599; doi:10.1038/srep34812)

GSF2 deletion increases lactic acid production by alleviating glucose repression in *Saccharomyces cerevisiae*

Seung-Ho Baek[1], Eunice Y. Kwon[1], Seon-Young Kim[2], and Ji-Sook Hahn[1*]

[1]School of Chemical and Biological Engineering, Institute of Chemical Processes, Seoul National University, 1 Gwanak-ro, Gwanak-gu, Seoul 08826, Republic of Korea

[2]Personalized Genomic Medicine Research Center, KRIBB, 125 Gwahag-ro, Yuseong-gu, Daejeon 34141, Republic of Korea

[*]Corresponding author: Phone: +82-2-880-9228

Fax: +82-2-888-1604

E-mail: hahnjs@snu.ac.kr

Supplementary Table S1. Primers used in this study

Primer	Sequence
Strain construction[a]	
d_GSF2 F	*GAGATCGGTGGACTTTGTTTTGATAGAGGGCGATTGCAAGC*CAGCTGAAGCTTCGTACGC
d_GSF2 R	*AATAAAAAAAAAGTCTGGATGGTAGTGTTTTGGTTTTACAA*GCATAGGCCACTAGTGGAT
d_SYN8 F	*CAGATCTCACGACAGCAAATAGATGCGTAAGCACACACGGT*CAGCTGAAGCTTCGTACGC
d_SYN8 R	*AACCAAACTTCGTATTCGAGCCTAAAAAACAGAATATAATG*GCATAGGCCACTAGTGGAT
d_STM1 F	*AAGTAGAAATAAACCAAGAAAGCATACACATTTTATTCTCA*CAGCTGAAGCTTCGTACGC
d_STM1 R	*GTTATTGGATTCTTTCAGTTGGAATTATTCATATATAAGGC*GCATAGGCCACTAGTGGAT
d_SIF2 F	*CAGAAACAAAAAAAAGGTAGGGAAGGCCCATCACACGGAAA*CAGCTGAAGCTTCGTACGC
d_SIF2 R	*AGAATGATAAAATTCATCTGTTTATGTACTGTACCTAGTTA*GCATAGGCCACTAGTGGAT
d_MIG1 F	*ACGAGAGTTGAGTATAGTGGAGACGACATACTACCATAGCC*CAGCTGAAGCTTCGTACGC
d_MIG1 R	*GTCTTTTGATTTATCTGCACCGCCAAAAACTTGTCAGCGTA*GCATAGGCCACTAGTGGAT
d_HXK2 F	*ATATAATTCTCCACACATAATAAGTACGCTAATTAAATAAA*CAGCTGAAGCTTCGTACGC
d_HXK2 F	*GGGCACCTTCTTGTTGTTCAAACTTAATTTACAAATTAAGT*GCATAGGCCACTAGTGGAT
c_GSF2 F	GGTCACTCCTTGTTCTTCT
c_GSF2 R	AGTAGATTCGTGAGGAATTG
c_SYN8 F	GCTGACTCATCTGCCACG
c_SYN8 R	GGTCCTTTTCTCCGGTAGG
c_STM1 F	GATATCATCGTTGCGTAGAG
c_STM1 R	CAAACAAACTACACGCTTGC
c_SIF2 F	CATGGAGCGGAACTTAGC
c_SIF2 R	GCTCATTGTCTTCTCTCATCG
c_MIG1 F	GTAAAGGCCCTGCGGCGCTT
c_MIG1 R	TTTTAGGGGGCCACAATAAA
c_HXK2 F	AATTTTCCACGACGACGACG
c_HXK2 R	TGAACAATAAATACGAAATC
Plasmid construction[b]	
GSF2 F	GGCGGGATCCATGGAGATTTACATTAGACTTAACG
GSF2 R	GGCGGTCGACTTAATTAGATTTTTTCAAATCATTCTTTTTT
SYN8 F	GGCGGGATCCATGGATGTGTTGAAGCTGG
SYN8 R	GGCGGTCGACTTATAATACTAATAGAAGCAACAGGAGCA
STM1 F	GGCGGGATCCATGTCCAACCCATTTGATTTG
STM1 R	GGCGGTCGACTTAAGCCAAAGATGGCAAGT
SIF2 F	GGCGGGATCCATGAGTATAACAAGTGAAGAACTAAAC
SIF2 R	GGCGGTCGACTTATATGGCTACAACTGAACCT

qRT-PCR	
q_ACT1 F	GCCGAAAGAATGCAAAAGGA
q_ACT1 R	TAGAACCACCAATCCAGACGG
q-COX6 F	CGCACATGATGAAGAAACCTTTGAGG
q-COX6 R	CCTCAAAGCTTTTTCAATAACAGCAGG
q_HXT1 F	CCATTAAGAGTCAAATCC
q_HXT1 R	GTAAGCGAAAACCATACA
q_NDI1 F	AAGTAGCGCACCAAGAGGC
q_NDI1 R	CGTATGGTTGCAATGGCCC
q_SDH1 F	GCTGACACTTTACAGCCTGGG
q_SDH1 R	ACTGCAGTAATGTTCCGAACACC
q_SUC2 F	TCCTGAAGAATATTTGAGAATGGGTT
q_SUC2 R	TCCAAGATGTTTTGATCCAGTAGGC

[a] The sequences homologous to target site are shown in italic.

[b] Restriction enzyme sites are underlined.

Supplementary Figure S1. pH changes of medium during the fermentation of LA-producing strain (JHY5210) and control strain (JHY5160). Each strain was cultured in 20 mL YPD medium containing 50 g/L glucose in a 100 mL flask. Residual glucose concentrations (a), production levels of D-LA (b) and acetate (c), and pH level (d) in the culture medium were monitored. Error bars indicate standard deviations of three independent experiments.

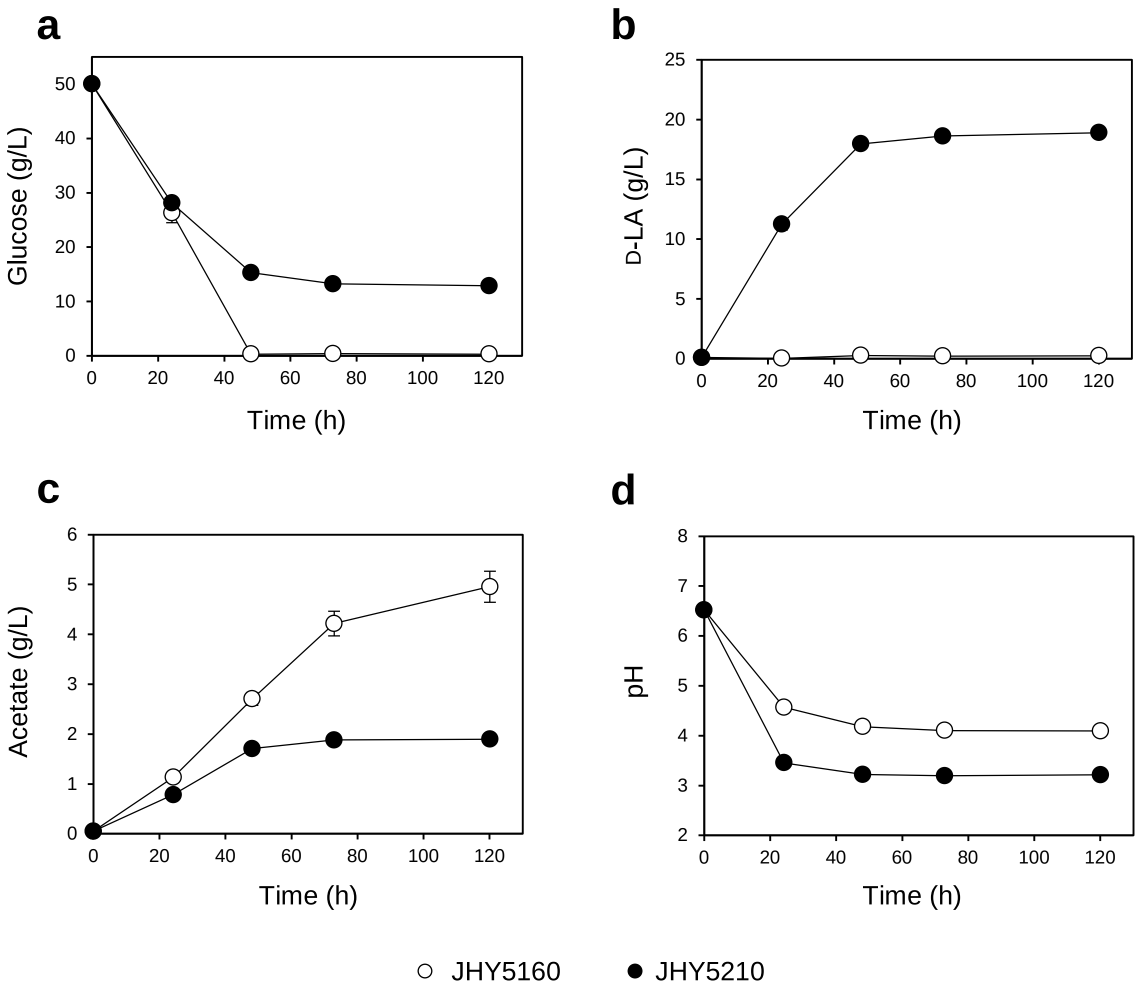

Supplement: Supplementary Information [file srep34812-s1.pdf]
